# Immunomodulation of CXCL10 Secretion by Hepatitis C Virus: Could CXCL10 Be a Prognostic Marker of Chronic Hepatitis C?

**DOI:** 10.1155/2019/5878960

**Published:** 2019-08-08

**Authors:** Silvia Martina Ferrari, Poupak Fallahi, Ilaria Ruffilli, Giusy Elia, Francesca Ragusa, Sabrina Rosaria Paparo, Armando Patrizio, Valeria Mazzi, Michele Colaci, Dilia Giuggioli, Clodoveo Ferri, Alessandro Antonelli

**Affiliations:** ^1^Department of Clinical and Experimental Medicine, University of Pisa, Pisa, Italy; ^2^Department of Translational Research and of New Technologies in Medicine and Surgery, University of Pisa, Pisa, Italy; ^3^Internal Medicine Unit, Cannizzaro Hospital, Department of Clinical and Experimental Medicine, University of Catania, Catania, Italy; ^4^Rheumatology Unit, Azienda Ospedaliero-Universitaria di Modena, University of Modena and Reggio Emilia, Modena, Italy

## Abstract

Chemokine (C-X-C motif) ligand (CXCL)10 and other CXCR3 chemokines are involved in the pathogenesis of acute and “chronic hepatitis C virus (HCV) infection” (CHC). Here, we review the scientific literature about HCV and CXCL10. The combination of circulating CXCL10 and single nucleotide polymorphisms (SNPs) in IL-28B can identify patients with acute HCV infection most likely to undergo spontaneous HCV clearance and those in need of early antiviral therapy. In CHC, the HCV and intrahepatic interferon- (IFN-) *γ* drive a raised CXCL10 expression by sinusoidal endothelium and hepatocytes, thereby inducing the recruitment of CXCR3-expressing T cells into the liver; thus, CXCL10 plays an important role in the development of necroinflammation and fibrosis. Increased CXCL10 was significantly associated with the presence of active vasculitis in HCV-associated cryoglobulinemia, or with autoimmune thyroiditis in CHC. Pretreatment CXCL10 levels are predictive of early virological response and sustained virological response (SVR) to IFN-*α* and ribavirin and may be useful in the evaluation of candidates for therapy. The occurrence of SNPs adjacent to IL-28B (rs12979860, rs12980275, and rs8099917), and CXCL10 below 150 pg/mL, independently predicted the first phase viral decline and rapid virological response, which in turn independently predicted SVR. Directly acting antiviral agents-mediated clearance of HCV is associated with the loss of intrahepatic immune activation by IFN-*α*, associated by decreased levels of CXCL10. In conclusion, CXCL10 is an important marker of HCV clearance and successful therapy in CHC patients. Whether CXCL10 is a novel therapeutic target in CHC will be evaluated.

## 1. Introduction

Hepatitis C is an infectious disease caused by the hepatitis C virus (HCV), affecting 150–200 million people worldwide [1]. Hepatitis C infection causes acute symptoms in 15% of cases. The infection resolves spontaneously in 10% of patients (more frequently in young and females) [2]. Approximately 85% of those HCV-exposed develop a chronic infection [3, 4]. HCV causes 27% of cirrhosis cases and 25% of hepatocellular carcinoma (HCC), worldwide [5–7].

About 38–76% of CHC patients develop at least one extrahepatic manifestation (EHM) [8]. The term “HCV syndrome” involves hepatic and extrahepatic disorders, and among them, mixed cryoglobulinemic vasculitis can be considered the pathophysiological prototype [9], involving various organs and systems (joints, kidney, nervous system, skin, and eyes). The infected extrahepatic tissues can function as a reservoir for HCV [9] that replicates and expresses viral proteins in extrahepatic tissues, contributing to EHMs. HCV avoids immune elimination, causing the accumulation of circulating immunocomplexes, and autoimmune phenomena [10].

EHMs include autoimmune disorders [11], such as MC [12] and Sjogren's syndrome, and endocrinological disorders, such as autoimmune thyroid disorders and type 2 diabetes [13], and range from mild or moderate manifestations (peripheral neuropathy, arthralgia, and sicca syndrome) to severe, life-threatening complications (neoplastic conditions and vasculitis) [14, 15].

In HCV infection, cytokines and chemokines take part in viral control and liver damage. Chemokine (C-X-C motif) ligand (CXCL)10 and the other CXCR3 chemokines participate in the pathogenesis of acute and “chronic hepatitis C virus (HCV) infection” (CHC).

## 2. Cytokines and Chemokines

Cytokines are small proteins, secreted by different types of cells: fibroblasts, immune cells (macrophages, B lymphocytes, and T lymphocytes), endothelial cells, and different epithelial cells. Upon binding to specific receptors, cytokines modulate the balance between cell-based and humoral immune responses and have a key role in host responses to infection, inflammation, immune responses, trauma, cancer, and sepsis [16, 17].

Chemokines are chemotactic cytokines of about 8-10 kDa in size. On the basis of the presence of 4 cysteine residues in conserved locations, chemokines are classified into 4 subfamilies: CXC (*α*), CC (*β*), C (*γ*), and CX3C (*δ*). Their receptors are CXCR, CCR, CR, and CX3CR, respectively.

IFN-*γ*-induced protein 10 (*IP-10*)/chemokine (C-X-C motif) ligand (CXCL)10 belongs to the ELR−CXC chemokine family [18], and it is secreted upon IFN-*γ* stimulation by different cell types (endothelial cells, keratinocytes, fibroblasts, monocytes, and T lymphocytes) [19]; also, proinflammatory cytokines (IFN-*α*, IFN-*β*, and TNF-*α* [20, 21]) are able to induce its secretion.

CXCL10 exerts its biological effects principally through CXCR3 [22], and it behaves as a chemoattractant for leukocytes, particularly Th1 lymphocytes [23].

The determination of high CXCL10 levels in peripheral liquids is believed to be a marker of host Th1-orientated immune response. Activated Th1 lymphocytes cause an increased IFN-*γ* and TNF-*α* production that stimulates the target cells to secrete CXCL10, thus perpetuating the immune cascade [24, 25].

An elevated tissue and/or cellular expression of CXCL10 is shown in organ-specific autoimmune diseases (including type 1 diabetes [26], Graves' disease [27] or Graves' ophthalmopathy [27], and AT [28–30]) or systemic rheumatological disorders (systemic lupus erythematosus, rheumatoid arthritis, systemic sclerosis [31–34], psoriasis [31] or psoriatic arthritis [35], and sarcoidosis [31, 36]).

Different neoplastic disorders show the involvement of the Th1 immune response [37–41], too.

Moreover, CXCL10 is involved in the pathogenesis and progression of infectious diseases [42] (i.e., Mycobacterium tuberculosis [43]) or HCV-related autoimmune disorders [10, 15, 44–46], sepsis [47], or HIV [48].

Here, we review the scientific literature about HCV infection and CXCL10 [given the complexity of the published data on CXCL10 with other infections, the coinfections (for example, HCV/HIV coinfection) will be excluded].

## 3. CXCL10 and Acute HCV Infection (Table 1)

During acute HCV infection, CXCL9-10-11 induction started 38-53 days and peaked 72-83 days after virus acquisition; a similar pattern was observed for alanine aminotransferase (ALT) levels. The increase of CXCR3-associated chemokines, in the late phase of acute HCV infection, leads to hypothesize a role for cellular immune responses in chemokine secretion [49].

In six chimpanzees infected with HCV, chemokine induction correlated with intrahepatic type I IFN responses *in vivo* and was stopped by the neutralization of antibodies against IFN-*β in vitro* [50].

A study evaluated IL-28B single nucleotide polymorphisms (SNPs) rs12979860 and rs8099917, together with serum CXCL10 levels, reporting that the frequency of IL-28B rs12979860 C/C genotype was higher in patients with spontaneous clearance than those without [51]. The CXCL10 median level was lower among patients with acute HCV infection and spontaneous clearance (764 pg/mL) than those without spontaneous clearance (1481 pg/mL). Considering also the data on CXCL10 levels, the capability of the IL-28B rs12979860 C/C of identifying patients more prone to have spontaneous clearance (83% of those who had a CXCL10 level < 540 pg/mL and 32% of whom with CXCL10 > 540 pg/mL) increased. Combining circulating CXCL10 and SNPs in IL-28B, patients with acute HCV infection who are more prone to have clearance spontaneously, and those who need an early antiviral therapy, can be identified [51]. These results were confirmed by other studies [52, 53].

Moreover, truncated CXCL10 is associated with the lack of spontaneous clearance of acute HCV infection, providing evidence of chemokine antagonism during acute HCV infection [54].

The last study reported that CXCR3 and chemokine receptor (CCR)2 were overexpressed on HCV-specific CD8+CCR7+CD45RO+ (central memory) cells. These results suggested that CXCR3-mediated signals accumulate HCV-specific CD8+ memory T cells in the infected liver [55].

## 4. CXCL10 and Chronic CHC (Table 2)

High levels of CXCR3 receptors are expressed by the lymphocytes that infiltrate a hepatitis C-infected liver [56]. CXCL10 and monokine-induced by IFN-*γ* (Mig)/CXCL9 are upregulated on sinusoidal endothelium. CXCL10 and CXCL9 are secreted by human IFN-*γ*+TNF-*α*-stimulated hepatic sinusoidal endothelial cells *in vitro*, suggesting that in CHC the production of IFN-*γ* in the liver causes the raised CXCL10 and CXCL9 expression and recruits CXCR3-expressing T cells in the hepatic lobule [56]. CXCR3 ligands are responsible for regional localization of specific lymphocyte subsets in the HCV-infected liver [57–59].

Another paper showed that in CHC (but not in other liver disorders), CXCL10 expression was highly correlated with the total transcripts of IFN-*γ* [60].

HCV can induce CXCL10 in hepatocytes, taking part in the pathogenesis of CHC [61].

Another paper reported that CXCL9 and CXCL10 expression in human hepatocyte-derived cells is upregulated by NS5A and core proteins, alone, or in combination, with cytokines [62].

Other papers confirmed the induction of CXCL10 release by HCV in hepatocytes [63, 64], involving TLR2 and CD44 [65]. CXCL10 and its noncognate receptor, TLR4, induce a proapoptotic signaling cascade for hepatocytes during liver injury [66].

To understand the molecular pathogenesis of the first stage of liver fibrosis (F1-CH-C) in hepatitis C patients, the mRNA expression of 240 selected genes in liver tissue with F1-CH-C was evaluated, compared to the normal liver (NL) [67]. The principal alterations in gene expression affected especially the IFN-regulated transcriptional network, such as IFN-*α*/*β*-inducible genes (STAT1, STAT2, etc.) and IFN-*γ*-inducible genes (CXCL9, CXCL10, and CXCL11) [67]. Among the IFN-inducible genes, from fibrosis stage F1 to fibrosis stage F4 (in particular the transition F1-CH-C to F2-CH-C), only the mRNA levels of IFN-*γ*-inducible genes increased at the limit of the statistical significance, while the expression of the IFN-*α*/*β*-inducible genes did not change [67].

A further study showed that in the liver CXCL10 mRNA expression was significantly associated with lobular necroinflammatory grade and HCV genotype 1 [68]. CXCL10 expression increased strongly with a higher stage of fibrosis compared to CXCL9 and CXCL11. The reported data suggested that in CHC, CXCL10 could be determinant in the development of necroinflammation and fibrosis in the liver [68].

Moreover, patients with advanced fibrosis had significantly increased CXCL10 plasma levels. The reported data suggested that in patients with HCV genotype 1 CXCR3-associated chemokines are promising noninvasive markers of hepatic fibrosis [69–71].

After liver transplantation (LT) for HCV infection, the recurrence of liver fibrosis can lead to graft loss and patient mortality [72]. One, 3, 5, and 7 years after LT, routine protocol biopsy was conducted, to evaluate the graft inflammation and fibrosis. In the first year upon LT, CXCL10 levels were highly associated with early fibrosis recurrence, regardless of other risk confounders, suggesting that after LT, CXCL10 is an independent biomarker of recurrent significant fibrosis for HCV infection [72].

Other papers, too, reported that in CHC, CXCL10 can be considered a marker of liver fibrosis [73–75].

Moreover, it has been shown that HCV-induced CXCL10 can lead to a raised hepatocyte turnover and the development of cirrhosis, fibrosis, and HCC [38].

In many HCV patients, circulating CXCL10 is enzymatically processed to produce a CXCL10 antagonist form, introducing a role for chemokine antagonism during HCV infection [76–78].

Patients with seronegative “occult HCV infection” (OCI) are anti-HCV and serum HCV-RNA negative but have viral RNA in the liver and altered levels of liver enzymes [79]. A study examined if the rs12979860 polymorphism of IL-28B and serum CXCL10 levels are different between chronic and seronegative OCI. The IL-28B CC genotype was significantly more prevalent in seronegative OCI (52.5%) than in CHC (24.6%; *P* < 0.0001) or healthy controls (32.5%; *P* = 0.039). Patients with seronegative OCI had significantly lower mean serum CXCL10 levels than CHC patients [79].

CXCL10 single nucleotide polymorphism rs1439490 G/G was more prevalent in OCI patients (90.3%) than in CHC patients (74.8%; *P* = 0.008), suggesting that CXCL10 rs1439490 G/G is positively associated with OCI in HCV [80].

CHC is associated with HCV-EHM. Several studies demonstrated elevated CXCL10 and TNF-*α* circulating levels in patients with HCV-associated cryoglobulinemia (MC+HCV) and raised CXCL10 significantly associated with the presence of active vasculitis [81], particularly in the presence of AT [82, 83].

## 5. HCV Therapy and CXCL10

In the first study, CXCL10 serum levels were significantly (*P* < 0.02) higher in patients with chronic hepatitis C (509.8 ± 365.4 pg/mL, mean ± s.d.) than in healthy controls (30.8 ± 20.0 pg/mL, mean ± s.d.). Serum CXCL10 was gradually reduced by IFN therapy, and after the successful treatment of CHC, circulating CXCL10 reached the same levels of healthy controls [59, 84].

In 29 HCV genotype 1-infected patients, CXCL10 and CXCL9 were high and decreased after an antiviral therapy with a good outcome [85]. The CXCL10 level measured before starting the antiviral treatment was greater in patients with HCV who then became nonresponders (NR) to therapy (*P* = 0.002), indicating that CXCL10 plasma levels can predict nonresponsiveness or responsiveness to antiviral therapy with pegylated IFN (PEG-IFN), in the presence/absence of ribavirin (RBV) [85].

CHC patients treated with PEG-IFN with sustained viral response (SVR) had significantly lower pretreatment circulating CXCL10 than NR patients (332.4 vs. 476.8 pg/mL). A significant decrease of serum CXCL10 was shown in patients with SVR but not in NR. Pretreatment serum CXCL10 levels and baseline viral load were reported to be predictive factors of SVR in HCV genotype 1 patients [86].

Another study investigated circulating CXCL10 in 265 HCV-infected patients, before, during, and after PEG-IFN-*α* 2a plus RBV treatment [87]. CXCL10 levels declined after the therapy, remaining low in patients with a SVR. On the contrary, at the end of the therapy, CXCL10 plasma levels rebounded in patients who had detectable HCV RNA. Using cut-off CXCL10 levels of 150 and 600 pg/mL to predict a SVR in HCV genotype 1-infected patients gave a specificity and sensitivity of 81% and 95%, respectively [87].

Another study reported significantly lower CXCL10 levels in patients with a rapid viral response (RVR) (*P* < 0.0001) and in patients obtaining a SVR (*P* = 0.0002) [88]. In multivariate logistic regression analyses, a low CXCL10 value independently predicted RVR and SVR. A baseline cut-off CXCL10 value of 600 pg/mL evidenced a negative predictive value (NPV) of 79% for patients with HCV genotype 1, similar to the one obtained using a reduction in HCV-RNA by at least 2 logs after 12 weeks of therapy (NPV 86%) [88].

Thyroid autoimmunity is a frequent side effect of IFN-*α* therapy for CHC [89, 90].

By pooling data about HCV-positive patients (with CHC or HCVAb positivity) and taking as control, the total of healthy subjects, HBV-infected patients, and sera negative for HCVAb, a significant increase of the prevalence of thyroid autoimmune disorders (odds ratio [OR] = 1.6; 95% confidence interval [CI] = 1.4–1.9) and of hypothyroidism (OR = 2.9; 95%CI = 2.0–4.1) has been described [90].

Both thyroid autoimmunity and dysfunction are common side effects of IFN-*α* therapy for CHC, being thyroid autoantibodies the main predisposing factor for the development of thyroid dysfunctions. A paper evaluated prospectively the thyroid status in patients in treatment with 2 distinct preparations of recombinant IFNs [91]. Most patients developing thyroid autoimmunity during the IFN treatment showed a destructive thyroiditis in short temporal relationship to the appearance of thyroid autoantibodies [91].

Pretreatment mean circulating CXCL10 was significantly higher in patients who did not develop thyroid antibody positivity or dysfunctions (Group I) compared to those who showed the appearance of serum thyroid antibodies and later clinically overt thyroid dysfunction (Group II). The two groups had different rates of favourable response to IFN-*α* treatment (33 and 90%, respectively) [89].

In a further study, CD8+ cells were increased in the liver and correlated with inflammation [92]. Patients with CHC showed high serum concentration of CXCL10/CCL3 (vs. healthy subjects). After PEG-IFN-*α*-2b plus RBV therapy, CCR5(high)/CXCR3(high)-expressing CD8+ cell frequency raised in the peripheral blood, while CXCL10/CCL3 serum concentration decreased. The obtainment of viral control was linked to a rise in CXCR3(high)-expressing CD8+ cells during the therapy [92].

Another study showed that in patients with CHC, low levels of intrahepatic and systemic CXCL10 predict a good first-phase decline of HCV RNA during PEG-IFN and RBV therapy [93].

In patients with a sufficient first response to PEG-IFN, RBV ameliorates the kinetics of the early response to therapy [94], and at 12 hours, serum CXCL10 was higher than that present in patients treated with PEG-IFN alone (7.6- vs. 3.8-fold; *P* = 0.01) [94].

Other papers agreed that pretreatment CXCL10 levels could predict SVR and the early virological response (EVR) to IFN-*α* therapy and RBV [95–99].

Serum CXCL10 levels were significantly lower in responders and are considered a predictor for SVR also in patients with HCV 4 genotype [100, 101].

In CHC patients treated with PEG-IFN and RBV, IL-28B gene polymorphisms are strongly associated with SVR [102]. CXCL10 was assessed in the pretreatment serum of 115 NR and 157 sustained responders. Mean CXCL10 was lower in sustained responders compared to NR (437 ± 31 vs. 704 ± 44 pg/mL, *P* < 0.001). The PPV of low CXCL10 levels (<600 pg/mL) for SVR was 69%, while the NPV of elevated CXCL10 levels (>600 pg/mL) was 67%. The three IL-28B genotypes (in particular rs12979860) CC, TT, and CT were reported in 30%, 21%, and 49% of patients, and the relative SVR rates were 87%, 39%, and 50%, respectively. The circulating CXCL10 levels within the IL-28B genotype groups gave more information regarding the probability of SVR (*P* < 0.0001). In patients with CT, CC, and TT genotypes, a higher SVR rate was reported in those with low CXCL10 levels versus those with high CXCL10 (CT, 64% versus 24%; CC, 89% versus 79%; and TT, 48% versus 20%). The combination of IL-28B genotype and pretreatment serum CXCL10 levels leads to a significantly higher predictive value to discriminate between SVR and nonresponse, especially in non-CC genotypes [102].

A second study confirmed that the occurrence of SNPs adjacent to IL-28B (rs12979860, rs12980275, and rs8099917), and CXCL10 below 150 pg/mL, independently predicted the first phase viral decline and RVR, which in turn independently predicted SVR [103].

Other papers, too, confirmed that CXCL10, in combination with IL-28B, could be a helpful marker to predict treatment failure in HCV patients [104–107].

The IFN-free direct antiviral agent (DAA) therapy has changed radically antiviral therapy in HCV infection, so that a successful treatment can be reached to practically all patients irrespective of their comorbidity [108]. Actually, 3 classes of DAAs are available, and they differ according to the specific viral protein they target: NS3/4A protease inhibitors (voxilaprevir, simeprevir, glecaprevir, grazoprevir, and paritaprevir); NS5A inhibitors (ombitasvir, ledipasvir, velpatasvir, elbasvir, pibrentasvir, and daclatasvir); and NS5B polymerase inhibitors that block replication of viral RNA (dasabuvir and sofosbuvir) [109]. During DAA treatment, the inhibition of HCV replication leads to reversal of NK cells and phenotypic and functional shifts of CD4 and CD8 T cells typical of CHC, triggering viral reactivation in patients with chronic hepatitis B and recurrence of HCC in some patients with earlier effective cancer treatment [109].

Several centers published the data obtained with novel DAA therapies in HCV-MCS [“mixed cryoglobulinemia syndrome” (MCS)], demonstrating a SVR in 297/313 (95%) patients, and 85% of them had a complete or partial clinical response of MCS symptoms. Patients with cryoglobulinemic glomerulonephritis had a good cure rate (94%). Seventeen/52 (33%) experienced full remission, and 15/52 (29%) had partial remission. Less than 5% of the patients with HCV-MCS treated with IFN-free DAA therapy required immunosuppression [110].

Recently, it has been also reported that baseline circulating CXCL10 can predict virological response in HCV genotype 1 CHC patients treated with telaprevir- (TVR-) based triple therapy, especially in patients with IL-28B risk allele [111]. CXCL10 was significantly more elevated in patients with (median, 570.06 pg/mL; range, 209.66–4297.62), than in those without (median, 394.64 pg/mL; range, 151.35–1146.43) (*P* = 0.001), advanced fibrosis (F3/F4). Furthermore, CXCL10 was significantly higher in patients with (median, 532.59 pg/mL; range, 151.35–1768.81) than in those without (median, 355.06 pg/mL; range, 155.53–4297.62) (*P* = 0.006) moderate/severe activity (METAVIR score A2/A3). CXCL10 correlated with liver fibrosis and inflammation [111].

Danoprevir is a powerful and selective DAA, targeting the protease activity of HCV NS3/4A [112]. During the treatment with danoprevir, changes in the CXCL10 plasma level were associated with categorical changes in HCV RNA concentration at days 7 and 14. Hepatic inflammation can be reduced by an effective treatment with a DAA and patients with an elevated baseline CXCL10 level have a higher first-phase HCV RNA decline during treatment with an NS3/4A protease inhibitor [112].

Actual therapeutic strategies are derived from the combination of PEG-IFN, RBV, and (only for patients with genotype 1 infection) a protease inhibitor. HCV treatment is developing fast from IFN-*α*-based therapies to IFN-*α*-free regimens consisting of DAAs that have an increased efficacy and tolerability in clinical trials. Virologic relapse after DAA therapy is a common cause of treatment failure, even if the reason of such relapse or whether some patients are more likely to recurrent viremia is still unknown. Circulating CXCL10 decreases with HCV clearance by DAA therapy.

Another study measured CXCL10 in 428 patients at baseline, week 1, and week 2 of oral treatment for HCV infection, suggesting that CXCL10 could be considered a surrogate marker of the rate of intracellular viral replication complex decay [113].

Peripheral and liver CXCL10 levels were evaluated in 15 patients administered with TVR/PEG-IFN/RBV, showing that CXCL10 identified very RVR in patients treated with DAAs [114].

It was investigated whether the elimination of HCV with DAAs normalizes the expression of IFN-stimulated genes and NK cell function, from the liver and blood of 13 HCV-infected patients who did not respond to treatment with PEG-IFN and RBV, during treatment with daclatasvir and asunaprevir [115]. DAA-mediated clearance of HCV was associated with the loss of intrahepatic immune activation by IFN-*α*, indicated by decreased levels of CXCL10 and CXCL11 and normalization of NK cell phenotype and function [115].

The treatment of CHC with DAAs results in a fast decline in viral load and circulating CXCL10 that were followed by a SVR [116].

Another paper evaluated the role of the immune system and miRNA levels in acquiring a SVR after DAA therapy in CHC patients with/without prior RG-101 (anti-miR-122) dosing [117]. Twenty-nine patients with HCV genotype 1 (*n* = 11), 3 (*n* = 17), or 4 (*n* = 1) were administered with sofosbuvir and daclatasvir ± ribavirin. Eighteen patients were treated earlier with RG-101. All patients had an SVR12. CXCL10 levels decreased during treatment, but remained high 24 weeks after the treatment in comparison to healthy subjects (median 53.82 and 39.4 pg/mL, *P* = 0.02). The reported data showed that the effective treatment of CHC patients with/without prior RG-101 dosing reduces broad immune activation and normalized miR-122 levels [117].

A paper by Yamagiwa et al. investigated the importance of pretreatment serum CXCL10 levels and IL-28B genotyping in predicting SVR to TVR-based triple therapy in patients with HCV 1 genotype [118]. The median CXCL10 levels were significantly lower in rapid virologic response (RVR) (343 pg/mL in RVR vs. 593 pg/mL in non-RVR patients, *P* < 0.01) or SVR (359 pg/mL in SVR vs. 566 pg/mL in non-SVR patients, *P* < 0.05) in the IL-28B non-TT genotype group. RVR rates were significantly lower in the group with higher serum CXCL10 levels (>450 pg/mL). In the non-TT IL-28B genotype group, RVR and SVR rates were significantly lower in the group with higher CXCL10 levels. SVR rates in the group with lower CXCL10 levels (<450 pg/mL) raised to 82% for those showing RVR, but decreased to 27% in the group with higher CXCL10 levels for those not showing RVR. The paper showed that the determination of serum CXCL10 levels before treatment could be useful to predict an effective virologic response to TVR-based triple therapy, particularly in patients with IL-28B non-TT genotype [118].

A study explored the dynamic gene expression profile of PBMCs collected from 27 HCV-infected patients undergoing DAA therapy [119]. The pretreatment expression level of IFN-induced protein 44 (IFI44) and CXCL10 correlated with the pretreatment expression level of IFN-*β*. After DAA treatment, a significant decrease (*P* < 0.05) in the expression levels of IFN-*β*, IFI44, and CXCL10 was observed in the PBMCs. These data showed that pretreatment activation of IFN-*β* response is rapidly normalized after DAA treatment [119].

## 6. Conclusion

CXCL10 and the other CXCR3 chemokines are chemoattractants for leukocytes, especially Th1 lymphocytes, and are involved in the pathogenesis of acute and “chronic hepatitis C virus (HCV) infection” (CHC).

The combination of the serum level of CXCL10 and SNPs in IL-28B can identify patients with acute HCV infection who are most likely to have clearance spontaneously and those in need of early antiviral therapy.

In CHC, the HCV and intrahepatic production of IFN-*γ* drive a raised CXCL10 and CXCL9 expression by sinusoidal endothelium, and by hepatocytes, and thereby promote the continuing recruitment of CXCR3-expressing T cells into the hepatic lobule. CXCL10 plays a key role in the development of necroinflammation and fibrosis in the liver parenchyma in CHC; furthermore, plasma levels of CXCL10 are significantly higher in patients with advanced fibrosis. In MC+HCV patients, increased CXCL10 levels were significantly associated with the presence of active vasculitis. Furthermore, in CHC and MC patients, CXCL10 circulating levels were higher in those with associated AT.

After the successful IFN therapy of CHC, the circulating CXCL10 levels declined to the same level as in a healthy volunteer. Pretreatment CXCL10 levels were predictive of both EVR and SVR to IFN-*α* and RBV and may be useful to evaluate candidates for therapy. The occurrence of SNPs adjacent to IL-28B (rs12979860, rs12980275, and rs8099917), and low circulating CXCL10, independently predicted the first phase viral decline and RVR, which in turn independently predicted SVR.

Actual therapeutic strategies are derived from the combination of PEG-IFN, RBV, and (only for patients with genotype 1 infection) a protease inhibitor (as telaprevir or boceprevir). Several molecules are under clinical evaluation for their potential to bypass the disadvantages of the actual available treatments [120].

The abovementioned studies underline the importance of CXCL10 as a marker of HCV clearance and successful therapy in CHC patients.

The above reported data indicate that in HCV-infected patients, antibodies able to block the interaction between CCR5/CXCR3 and their ligands could decrease chronic liver inflammation. Since HCV is not directly cytopathic, if the migration of nonspecific T cells to the infected liver is impaired, liver damage will be reduced. This strategy could be evaluated in patients without a SVR after current standard treatment [71].

Virtually, some chemokines and their receptors are prognostic tools to predict anti-HCV treatment responses, and if this could be confirmed, these predictors could be added to daily clinical practice. Moreover, the blocking of chemokines (CXCL10) and chemokine receptors (CXCR3) is a therapeutic strategy to be evaluated in the future for nonresponders to current anti-HCV therapy [71].

## Figures and Tables

**Table 1:** CXCL10 and acute HCV infection.

CXCL10 and acute HCV infection	References
Increase of CXCR3-associated chemokines, during the late phase of acute HCV infection	[49]
An *in vivo* study showed that despite an early-stage induction of chemokines, the intrahepatic lymphocytic infiltrate increased not earlier than 8 weeks after HCV infection	[50]
CXCL10 serum levels and SNPs in IL-28B can identify patients with acute hepatitis C who are more prone to undergo spontaneous clearance and those in need of early antiviral therapy	[51]
Truncated CXCL10 is associated with failure to obtain spontaneous clearance of acute hepatitis C infection	[54]
Both CXCR3 and CCR2 were overexpressed on HCV-specific CD8+CCR7+CD45RO+ (central memory) cells	[55]

**Table 2:** CXCL10 and chronic CHC.

CXCL10 and chronic CHC	References
The intrahepatic production of IFN-*γ* causes the raised CXCL10 and CXCL9 expression and recruits CXCR3-expressing T cells in the hepatic lobule.	[56]
CXCR3 ligands are responsible for regional localization of specific lymphocyte subsets in the HCV-infected liver.	[57]
Increased expression, in chronic HCV infection, of IFN-*α*/*β*-inducible antiviral MxA gene; gene encoding IFN-*α*/*β*-inducible p44; gene encoding IFN-*α*/*β*/*γ*-inducible IFI-56 K.	[58]
Hepatocytes in inflammatory areas produce CXCL10 either in autoimmune liver diseases or in chronic viral hepatitis.	[59]
Hepatic inflammatory activity was associated more strongly with IFN-*γ* than with CXCL10.	[60]
CXCL10 may be induced by HCV within hepatocytes, resulting to be an important factor in the pathogenesis of CHC.	[61]
CXCL10 and CXCL9 gene expression in cultured human hepatocyte-derived cells is upregulated by NS5A and core proteins, alone or in combination with cytokines.	[62]
Hepatocytes in CHC patients participate in CXCL10 production involving TLR2 and CD44.	[65]
CXCL10 and its noncognate receptor, TLR4, are proapoptotic signaling cascades for hepatocytes during liver injury.	[66]
Nonparenchymal hepatic cells and immune effector cells were recruited in the site of infection by CXCL10. These cells by secreting type I, II, and III IFNs amplify CXCL10 response during the later stages of acute HCV infection.	[63]
Investigations about the mechanisms of CXCL10 induction in hepatocytes by several factors (such as NF-*κ*B and IRF3). Perpetual inflammation and viral persistence could be arisen because of viral proteins that antagonize with these factors and then interfere with the induction of CXCL10.	[64]
In the first stage of liver fibrosis, the principal alterations in gene expression affected in particular the IFN-regulated transcriptional network, such as IFN-*α*/*β*-inducible genes (STAT1, STAT2, ISGF3G/IRF9, IFI27, G1P3, G1P2, OAS2, and MX1) and IFN-*γ*-inducible genes (CXCL9, CXCL10, and CXCL11).	[67]
CXCL10 mRNA expression levels were significantly associated with lobular necroinflammatory grade and HCV genotype 1.	[68]
Patients with advanced fibrosis had significantly increased CXCL10 plasma levels.	[69]
CXCR3 chemokines are the most strongly expressed chemokines in CHC and presumably have a key role in positioning T cells in the liver.	[70, 71]
CXCL10 is an independent biomarker of the recurrence of significant fibrosis after liver transplantation for HCV infection.	[72]
Studies reported that in CHC, CXCL10 can be considered a marker of liver fibrosis.	[73–75]
HCV-induced CXCL10 can lead to a raised hepatocyte turnover and the development of cirrhosis, fibrosis, and HCC.	[37]
Circulating CXCL10 in many HCV patients is enzymatically processed to produce a CXCL10 antagonist form, introducing a role for chemokine antagonism during HCV infection.	[76–78]
Serum CXCL10 levels were significantly lower in patients with seronegative occult HCV infection than in patients with chronic hepatitis C.	[79]
The single nucleotide polymorphism CXCL10 rs1439490 G/G is positively associated with occult HCV infection in HCV.	[80]
High CXCL10 and TNF-*α* serum levels were observed in patients with hepatitis C-associated cryoglobulinemia (MC+HCV), and in particular, increased CXCL10 levels were significantly associated with the presence of active vasculitis.	[81]
In mixed cryoglobulinemia patients, circulating CXCL10 was higher in those with associated autoimmune thyroiditis.	[82]

## References

[1] Lemoine M., Thursz M. (2014). Hepatitis C, a global issue: access to care and new therapeutic and preventive approaches in resource-constrained areas. *Seminars in Liver Disease*.

[2] Maheshwari A., Ray S., Thuluvath P. J. (2008). Acute hepatitis C. *The Lancet*.

[3] Patel K., Muir A. J., McHutchison J. G. (2006). Diagnosis and treatment of chronic hepatitis C infection. *British Medical Journal*.

[4] Rosen H. R. (2011). Chronic hepatitis C infection. *The New England Journal of Medicine*.

[5] Calvaruso V., Craxì A. (2012). 2011 European Association of the study of the liver hepatitis C virus clinical practice guidelines. *Liver International*.

[6] Sherman M. (2005). Hepatocellular carcinoma: epidemiology, risk factors, and screening. *Seminars in Liver Disease*.

[7] Kohli A., Shaffer A., Sherman A., Kottilil S. (2014). Treatment of hepatitis C: a systematic review. *JAMA*.

[8] Sebastiani M., Giuggioli D., Colaci M. (2017). HCV-related rheumatic manifestations and therapeutic strategies. *Current Drug Targets*.

[9] Ferri C., Sebastiani M., Giuggioli D. (2015). Hepatitis C virus syndrome: a constellation of organ- and non-organ specific autoimmune disorders, B-cell non-Hodgkin’s lymphoma, and cancer. *World Journal of Hepatology*.

[10] Ferrari S. M., Fallahi P., Mancusi C. (2013). HCV-related autoimmune disorders in HCV chronic infection. *La Clinica Terapeutica*.

[11] Blackard J. T., Kong L., Huber A. K., Tomer Y. (2013). Hepatitis C virus infection of a thyroid cell line: implications for pathogenesis of hepatitis C virus and thyroiditis. *Thyroid*.

[12] Giuggioli D., Sebastiani M., Colaci M. (2017). Treatment of HCV-related mixed cryoglobulinemia. *Current Drug Targets*.

[13] Fabiani S., Fallahi P., Ferrari S. M., Miccoli M., Antonelli A. (2018). Hepatitis C virus infection and development of type 2 diabetes mellitus: systematic review and meta-analysis of the literature. *Reviews in Endocrine & Metabolic Disorders*.

[14] Ferri C., Ramos-Casals M., Zignego A. L. (2016). International diagnostic guidelines for patients with HCV-related extrahepatic manifestations. A multidisciplinary expert statement. *Autoimmunity Reviews*.

[15] Zignego A. L., Gragnani L., Piluso A. (2015). Virus-driven autoimmunity and lymphoproliferation: the example of HCV infection. *Expert Review of Clinical Immunology*.

[16] Antonelli A., Miccoli P., Derzhitski V. E., Panasiuk G., Solovieva N., Baschieri L. (1996). Epidemiologic and clinical evaluation of thyroid cancer in children from the Gomel region (Belarus). *World Journal of Surgery*.

[17] Steinke J. W., Borish L. (2006). 3. Cytokines and chemokines. *The Journal of Allergy and Clinical Immunology*.

[18] Zlotnik A., Yoshie O. (2012). The chemokine superfamily revisited. *Immunity*.

[19] Luster A. D., Ravetch J. V. (1987). Biochemical characterization of a gamma interferon-inducible cytokine (IP-10). *The Journal of Experimental Medicine*.

[20] Ohmori Y., Hamilton T. A. (1994). Cell type and stimulus specific regulation of chemokine gene expression. *Biochemical and Biophysical Research Communications*.

[21] Ohmori Y., Hamilton T. A. (1995). The interferon-stimulated response element and a kappa B site mediate synergistic induction of murine IP-10 gene transcription by IFN-gamma and TNF-alpha. *The Journal of Immunology*.

[22] Loetscher M., Gerber B., Loetscher P. (1996). Chemokine receptor specific for IP10 and mig: structure, function, and expression in activated T-lymphocytes. *The Journal of Experimental Medicine*.

[23] Romagnani P., Crescioli C. (2012). CXCL10: a candidate biomarker in transplantation. *Clinica Chimica Acta*.

[24] Antonelli A., Ferrari S. M., Giuggioli D., Ferrannini E., Ferri C., Fallahi P. (2014). Chemokine (C-X-C motif) ligand (CXCL)10 in autoimmune diseases. *Autoimmunity Reviews*.

[25] Antonelli A., Ferrari S. M., Frascerra S. (2011). Increase of circulating CXCL9 and CXCL11 associated with euthyroid or subclinically hypothyroid autoimmune thyroiditis. *The Journal of Clinical Endocrinology and Metabolism*.

[26] Antonelli A., Ferri C., Fallahi P. (2004). Type 2 diabetes in hepatitis C-related mixed cryoglobulinaemia patients. *Rheumatology*.

[27] Antonelli A., Ferrari S. M., Fallahi P. (2011). Cytokines (interferon-*γ* and tumor necrosis factor–*α*)-induced nuclear factor–*κ*B activation and chemokine (C-X-C motif) ligand 10 release in Graves disease and ophthalmopathy are modulated by pioglitazone. *Metabolism*.

[28] Miccoli P., Antonelli A., Iacconi P., Alberti B., Gambuzza C., Baschieri L. (1993). Prospective, randomized, double-blind study about effectiveness of levothyroxine suppressive therapy in prevention of recurrence after operation: result at the third year of follow-up. *Surgery*.

[29] Antonelli A., Ferri C., Fallahi P. (2004). Thyroid involvement in patients with overt HCV-related mixed cryoglobulinaemia. *QJM*.

[30] Antonelli A., Ferrari S. M., Frascerra S. (2011). Circulating chemokine (CXC motif) ligand (CXCL)9 is increased in aggressive chronic autoimmune thyroiditis, in association with CXCL10. *Cytokine*.

[31] Antonelli A., Ferrari S. M., Corrado A., Di Domenicantonio A., Fallahi P. (2015). Autoimmune thyroid disorders. *Autoimmunity Reviews*.

[32] Giuggioli D., Manfredi A., Colaci M., Manzini C. U., Antonelli A., Ferri C. (2013). Systemic sclerosis and cryoglobulinemia: our experience with overlapping syndrome of scleroderma and severe cryoglobulinemic vasculitis and review of the literature. *Autoimmunity Reviews*.

[33] Antonelli A., Ferri C., Fallahi P. (2008). Th1 and Th2 chemokine serum levels in systemic sclerosis in the presence or absence of autoimmune thyroiditis. *The Journal of Rheumatology*.

[34] Giuggioli D., Lumetti F., Colaci M., Fallahi P., Antonelli A., Ferri C. (2015). Rituximab in the treatment of patients with systemic sclerosis. Our experience and review of the literature. *Autoimmunity Reviews*.

[35] Antonelli A., Fallahi P., Sedie A. D. (2008). High values of alpha (CXCL10) and beta (CCL2) circulating chemokines in patients with psoriatic arthritis, in presence or absence of autoimmune thyroiditis. *Autoimmunity*.

[36] Fallahi P., Ferrari S. M., Ruffilli I. (2016). The association of other autoimmune diseases in patients with autoimmune thyroiditis: review of the literature and report of a large series of patients. *Autoimmunity Reviews*.

[37] Billottet C., Quemener C., Bikfalvi A. (2013). CXCR3, a double-edged sword in tumor progression and angiogenesis. *Biochimica et Biophysica Acta (BBA) - Reviews on Cancer*.

[38] Brownell J., Polyak S. J. (2013). Molecular pathways: hepatitis C virus, CXCL10, and the inflammatory road to liver cancer. *Clinical Cancer Research*.

[39] Rainczuk A., Rao J., Gathercole J., Stephens A. N. (2012). The emerging role of CXC chemokines in epithelial ovarian cancer. *Reproduction*.

[40] Verbeke H., Geboes K., Van Damme J., Struyf S. (2012). The role of CXC chemokines in the transition of chronic inflammation to esophageal and gastric cancer. *Biochimica et Biophysica Acta (BBA) - Reviews on Cancer*.

[41] Ben-Baruch A. (2006). The multifaceted roles of chemokines in malignancy. *Cancer and Metastasis Reviews*.

[42] Liu M., Guo S., Hibbert J. M. (2011). CXCL10/IP-10 in infectious diseases pathogenesis and potential therapeutic implications. *Cytokine & Growth Factor Reviews*.

[43] Chegou N. N., Heyckendorf J., Walzl G., Lange C., Ruhwald M. (2014). Beyond the IFN-*γ* horizon: biomarkers for immunodiagnosis of infection with *Mycobacterium tuberculosis*. *The European Respiratory Journal*.

[44] Coppola N., Marrone A., Pisaturo M. (2014). Role of interleukin 28-B in the spontaneous and treatment-related clearance of HCV infection in patients with chronic HBV/HCV dual infection. *European Journal of Clinical Microbiology & Infectious Diseases*.

[45] Coppola N., Gentile I., Pasquale G. (2013). Anti-HBc positivity was associated with histological cirrhosis in patients with chronic hepatitis C. *Annals of Hepatology*.

[46] Fallahi P., Ferri C., Ferrari S. M., Corrado A., Sansonno D., Antonelli A. (2012). Cytokines and HCV-related disorders. *Clinical and Developmental Immunology*.

[47] Chan T., Gu F. (2011). Early diagnosis of sepsis using serum biomarkers. *Expert Review of Molecular Diagnostics*.

[48] Yao H., Bethel-Brown C., Li C. Z., Buch S. J. (2010). HIV neuropathogenesis: a tight rope walk of innate immunity. *Journal of Neuroimmune Pharmacology*.

[49] Zeremski M., Hooker G., Shu M. A. (2011). Induction of CXCR3- and CCR5-associated chemokines during acute hepatitis C virus infection. *Journal of Hepatology*.

[50] Shin E.–. C., Park S.–. H., DeMino M. (2011). Delayed induction, not impaired recruitment, of specific CD8^+^ T cells causes the late onset of acute hepatitis C. *Gastroenterology*.

[51] Beinhardt S., Aberle J. H., Strasser M. (2012). Serum level of IP-10 increases predictive value of *IL28B* polymorphisms for spontaneous Clearance of Acute HCV infection. *Gastroenterology*.

[52] Grebely J., Feld J. J., Applegate T. (2013). Plasma interferon-gamma-inducible protein-10 (IP-10) levels during acute hepatitis C virus infection. *Hepatology*.

[53] Beinhardt S., Payer B. A., Datz C. (2013). A diagnostic score for the prediction of spontaneous resolution of acute hepatitis C virus infection. *Journal of Hepatology*.

[54] Riva A., Laird M., Casrouge A. (2014). Truncated CXCL10 is associated with failure to achieve spontaneous clearance of acute hepatitis C infection. *Hepatology*.

[55] Pirozyan M. R., Nguyen N., Cameron B. (2019). Chemokine-regulated recruitment of antigen-specific T-cell subpopulations to the liver in acute and chronic hepatitis C infection. *The Journal of Infectious Diseases*.

[56] Shields P. L., Morland C. M., Salmon M., Qin S., Hubscher S. G., Adams D. H. (1999). Chemokine and chemokine receptor interactions provide a mechanism for selective T cell recruitment to specific liver compartments within hepatitis C-infected liver. *The Journal of Immunology*.

[57] Nguyen N., de Esch C., Cameron B., Kumar R. K., Zekry A., Lloyd A. R. (2014). Positioning of leukocyte subsets in the portal and lobular compartments of hepatitis C virus-infected liver correlates with local chemokine expression. *Journal of Gastroenterology and Hepatology*.

[58] Patzwahl R., Meier V., Ramadori G., Mihm S. (2001). Enhanced expression of interferon-regulated genes in the liver of patients with chronic hepatitis C virus infection: detection by suppression-subtractive hybridization. *Journal of Virology*.

[59] Nishioji K., Okanoue T., Itoh Y. (2001). Increase of chemokine interferon-inducible protein-10 (IP-10) in the serum of patients with autoimmune liver diseases and increase of its mRNA expression in hepatocytes. *Clinical and Experimental Immunology*.

[60] Mihm S., Schweyer S., Ramadori G. (2003). Expression of the chemokine IP-10 correlates with the accumulation of hepatic IFN-*γ* and IL-18 mRNA in chronic hepatitis C but not in hepatitis B. *Journal of Medical Virology*.

[61] Harvey C. E., Post J. J., Palladinetti P. (2003). Expression of the chemokine IP-10 (CXCL10) by hepatocytes in chronic hepatitis C virus infection correlates with histological severity and lobular inflammation. *Journal of Leukocyte Biology*.

[62] Apolinario A., Majano P. L., Lorente R., Nunez O., Clemente G., Garcia-Monzon C. (2005). Gene expression profile of T-cell-specific chemokines in human hepatocyte-derived cells: evidence for a synergistic inducer effect of cytokines and hepatitis C virus proteins. *Journal of Viral Hepatitis*.

[63] Brownell J., Wagoner J., Lovelace E. S. (2013). Independent, parallel pathways to CXCL10 induction in HCV-infected hepatocytes. *Journal of Hepatology*.

[64] Brownell J., Bruckner J., Wagoner J. (2014). Direct, interferon-independent activation of the CXCL10 promoter by NF-*κ*B and interferon regulatory factor 3 during hepatitis C virus infection. *Journal of Virology*.

[65] Abe T., Fukuhara T., Wen X. (2012). CD44 participates in IP-10 induction in cells in which hepatitis C virus RNA is replicating, through an interaction with Toll-like receptor 2 and hyaluronan. *Journal of Virology*.

[66] Sahin H., Borkham-Kamphorst E., do O N. T. (2013). Proapoptotic effects of the chemokine, CXCL 10 are mediated by the noncognate receptor TLR4 in hepatocytes. *Hepatology*.

[67] Bièche I., Asselah T., Laurendeau I. (2005). Molecular profiling of early stage liver fibrosis in patients with chronic hepatitis C virus infection. *Virology*.

[68] Zeremski M., Petrovic L. M., Chiriboga L. (2008). Intrahepatic levels of CXCR3-associated chemokines correlate with liver inflammation and fibrosis in chronic hepatitis C. *Hepatology*.

[69] Zeremski M., Dimova R., Brown Q., Jacobson I. M., Markatou M., Talal A. H. (2009). Peripheral CXCR3-associated chemokines as biomarkers of fibrosis in chronic hepatitis C virus infection. *The Journal of Infectious Diseases*.

[70] Helbig K. J., Ruszkiewicz A., Lanford R. E. (2009). Differential expression of the CXCR3 ligands in chronic hepatitis C virus (HCV) infection and their modulation by HCV in vitro. *Journal of Virology*.

[71] Larrubia J. R., Benito-Martínez S., Calvino M., Sanz-de-Villalobos E., Parra-Cid T. (2008). Role of chemokines and their receptors in viral persistence and liver damage during chronic hepatitis C virus infection. *World Journal of Gastroenterology*.

[72] Berres M. L., Trautwein C., Schmeding M. (2011). Serum chemokine CXC ligand 10 (CXCL10) predicts fibrosis progression after liver transplantation for hepatitis C infection. *Hepatology*.

[73] Zeremski M., Dimova R., Astemborski J., Thomas D. L., Talal A. H. (2011). CXCL9 and CXCL10 chemokines as predictors of liver fibrosis in a cohort of primarily African-American injection drug users with chronic hepatitis C. *The Journal of Infectious Diseases*.

[74] Joshi D., Carey I., Foxton M. (2014). CXCL10 levels identify individuals with rapid fibrosis at 12 months post-transplant for hepatitis C virus and predict treatment response. *Clinical Transplantation*.

[75] Patel K., Remlinger K. S., Walker T. G. (2014). Multiplex protein analysis to determine fibrosis stage and progression in patients with chronic hepatitis C. *Clinical Gastroenterology and Hepatology*.

[76] Casrouge A., Decalf J., Ahloulay M. (2011). Evidence for an antagonist form of the chemokine CXCL10 in patients chronically infected with HCV. *The Journal of Clinical Investigation*.

[77] Charles E. D., Dustin L. B. (2011). Chemokine antagonism in chronic hepatitis C virus infection. *The Journal of Clinical Investigation*.

[78] Casrouge A., Bisiaux A., Stephen L. (2012). Discrimination of agonist and antagonist forms of CXCL10 in biological samples. *Clinical & Experimental Immunology*.

[79] Bartolomé J., Castillo I., Quiroga J. A., Carreño V. (2016). Interleukin-28B polymorphisms and interferon gamma inducible protein-10 serum levels in seronegative occult hepatitis C virus infection. *Journal of Medical Virology*.

[80] Wang X., Wang S., Liu Z. H. (2018). Regulatory polymorphism of CXCL10 rs1439490 in seronegative occult hepatitis C virus infection. *World Journal of Gastroenterology*.

[81] Antonelli A., Ferri C., Ferrari S. M. (2010). Serum concentrations of interleukin 1*β*, CXCL10, and interferon-*γ* in mixed cryoglobulinemia associated with hepatitis C infection. *The Journal of Rheumatology*.

[82] Antonelli A., Ferri C., Ferrari S. M. (2010). Interleukin-1*β*, C-x-C motif ligand 10, and interferon-gamma serum levels in mixed cryoglobulinemia with or without autoimmune thyroiditis. *Journal of Interferon & Cytokine Research*.

[83] Antonelli A., Fallahi P., Ferrari S. M. (2012). Circulating CXCL11 and CXCL10 are increased in hepatitis C-associated cryoglobulinemia in the presence of autoimmune thyroiditis. *Modern Rheumatology*.

[84] Itoh Y., Morita A., Nishioji K. (2001). Clinical significance of elevated serum interferon- inducible protein-10 levels in hepatitis C virus carriers with persistently normal serum transaminase levels. *Journal of Viral Hepatitis*.

[85] Butera D., Marukian S., Iwamaye A. E. (2005). Plasma chemokine levels correlate with the outcome of antiviral therapy in patients with hepatitis C. *Blood*.

[86] Diago M., Castellano G., García-Samaniego J. (2006). Association of pretreatment serum interferon *γ* inducible protein 10 levels with sustained virological response to peginterferon plus ribavirin therapy in genotype 1 infected patients with chronic hepatitis C. *Gut*.

[87] Romero A. I., Lagging M., Westin J. (2006). Interferon (IFN)–*γ*–inducible protein–10: association with histological results, viral kinetics, and outcome during treatment with pegylated IFN‐*α*2a and ribavirin for chronic hepatitis C virus infection. *The Journal of Infectious Diseases*.

[88] Lagging M., Romero A. I., Westin J. (2006). IP-10 predicts viral response and therapeutic outcome in difficult-to-treat patients with HCV genotype 1 infection. *Hepatology*.

[89] Rotondi M., Minelli R., Magri F. (2007). Serum CXCL10 levels and occurrence of thyroid dysfunction in patients treated with interferon-*α* therapy for hepatitis C virus-related hepatitis. *European Journal of Endocrinology*.

[90] Antonelli A., Ferri C., Fallahi P. (2006). Thyroid disorders in chronic hepatitis C virus infection. *Thyroid*.

[91] Mazziotti G., Sorvillo F., Stornaiuolo G. (2002). Temporal relationship between the appearance of thyroid autoantibodies and development of destructive thyroiditis in patients undergoing treatment with two different type-1 interferons for HCV-related chronic hepatitis: a prospective study. *Journal of Endocrinological Investigation*.

[92] Larrubia J. R., Calvino M., Benito S. (2007). The role of CCR5/CXCR3 expressing CD8^+^ cells in liver damage and viral control during persistent hepatitis C virus infection. *Journal of Hepatology*.

[93] Askarieh G., Alsiö Å., Pugnale P. (2010). Systemic and intrahepatic interferon-gamma-inducible protein 10 kDa predicts the first-phase decline in hepatitis C virus RNA and overall viral response to therapy in chronic hepatitis C. *Hepatology*.

[94] Feld J. J., Lutchman G. A., Heller T. (2010). Ribavirin improves early responses to peginterferon through improved interferon signaling. *Gastroenterology*.

[95] Moura A. S., Carmo R. A., Teixeira A. L., Teixeira M. M., da Costa Rocha M. O. (2011). Soluble inflammatory markers as predictors of virological response in patients with chronic hepatitis C virus infection treated with interferon-*α* plus ribavirin. *Memórias do Instituto Oswaldo Cruz*.

[96] Sixtos-Alonso M. S., Sánchez-Muñoz F., Sánchez-Ávila J. F. (2011). IFN-stimulated gene expression is a useful potential molecular marker of response to antiviral treatment with Peg-IFN*α* 2b and ribavirin in patients with hepatitis C virus genotype 1. *Archives of Medical Research*.

[97] Kurelac I., Lepej S. Z., Grgic I. (2012). Chemokine CXCL10 at week 4 of treatment predicts sustained virological response in patients with chronic hepatitis C. *Journal of Interferon & Cytokine Research*.

[98] Matsuura K., Watanabe T., Iijima S. (2014). Serum interferon-gamma-inducible protein-10 concentrations and *IL28B* genotype associated with responses to pegylated interferon plus ribavirin with and without telaprevir for chronic hepatitis C. *Hepatology Research*.

[99] Omran D., Hamdy S., Tawfik S., Esmat S., Saleh D. A., Zayed R. A. (2014). Association of interferon-*γ* inducible protein-10 pretreatment level and sustained virological response in HCV-positive Egyptian patients. *Annals of Clinical and Laboratory Science*.

[100] Al-Ashgar H. I., Khan M. Q., Helmy A. (2013). Relationship of interferon-*γ*-inducible protein-10 kDa with viral response in patients with various heterogeneities of hepatitis C virus genotype-4. *European Journal of Gastroenterology & Hepatology*.

[101] Khalifa K. A., Radwan W. M., Attallah K. M., Hosney H., Eed E. M. (2014). Pretreatment serum interferon gamma inducible protein-10 as biomarker of fibrosis and predictor of virological response in genotype 4 hepatitis C virus infection. *Acta Gastro-Enterologica Belgica*.

[102] Darling J. M., Aerssens J., Fanning G. (2011). Quantitation of pretreatment serum interferon-*γ*-inducible protein-10 improves the predictive value of an *IL28B* gene polymorphism for hepatitis C treatment response. *Hepatology*.

[103] Lagging M., Askarieh G., Negro F. (2011). Response prediction in chronic hepatitis C by assessment of IP-10 and *IL28B*-related single nucleotide polymorphisms. *PLoS One*.

[104] Fattovich G., Covolo L., Bibert S. (2011). IL28B polymorphisms, IP-10 and viral load predict virological response to therapy in chronic hepatitis C. *Alimentary Pharmacology & Therapeutics*.

[105] Albert M. L., Casrouge A., Chevaliez S. (2011). Interferon induced protein 10 remains a useful biomarker of treatment failure in patients stratified for the interleukin-28B rs12979860 haplotype. *Hepatology*.

[106] Payer B. A., Reiberger T., Aberle J. (2012). IL28B and interferon-gamma inducible protein 10 for prediction of rapid virologic response and sustained virologic response in HIV-HCV-coinfected patients. *European Journal of Clinical Investigation*.

[107] Derbala M., Rizk N. M., al-Kaabi S. (2013). The predictive value of IL28B rs12979860, rs11881222 and rs8099917 polymorphisms and IP-10 in the therapeutic response of Egyptian genotype 4 patients. *Virology*.

[108] Spengler U. (2018). Direct antiviral agents (DAAs) - a new age in the treatment of hepatitis C virus infection. *Pharmacology & Therapeutics*.

[109] Urbanowicz A., Zagożdżon R., Ciszek M. (2019). Modulation of the immune system in chronic hepatitis C and during antiviral interferon-free therapy. *Archivum Immunologiae et Therapiae Experimentalis*.

[110] Rutledge S. M., Chung R. T., Sise M. E. (2018). Treatment of hepatitis C virus infection in patients with mixed cryoglobulinemic syndrome and cryoglobulinemic glomerulonephritis. *Hemodialysis International*.

[111] Nishikawa H., Enomoto H., Nasu A. (2014). Clinical significance of pretreatment serum interferon-gamma-inducible protein 10 concentrations in chronic hepatitis C patients treated with telaprevir-based triple therapy. *Hepatology Research*.

[112] Schaefer C. J., Kossen K., Lim S. R. (2011). Danoprevir monotherapy decreases inflammatory markers in patients with chronic hepatitis C virus infection. *Antimicrobial Agents and Chemotherapy*.

[113] Lin J. C., Habersetzer F., Rodriguez-Torres M. (2014). Interferon *γ*-induced protein 10 kinetics in treatment-naive versus treatment-experienced patients receiving interferon-free therapy for hepatitis C virus infection: implications for the innate immune response. *The Journal of Infectious Diseases*.

[114] Zeremski M., Dimova R. B., Benjamin S., Penney M. S., Botfield M. C., Talal A. H. (2015). Intrahepatic and peripheral CXCL10 expression in hepatitis C virus-infected patients treated with telaprevir, pegylated interferon, and ribavirin. *The Journal of Infectious Diseases*.

[115] Serti E., Chepa-Lotrea X., Kim Y. J. (2015). Successful interferon-free therapy of chronic hepatitis C virus infection normalizes natural killer cell function. *Gastroenterology*.

[116] Meissner E. G., Kohli A., Higgins J. (2017). Rapid changes in peripheral lymphocyte concentrations during interferon-free treatment of chronic hepatitis C virus infection. *Hepatology Communications*.

[117] van der Ree M. H., Stelma F., Willemse S. B. (2017). Immune responses in DAA treated chronic hepatitis C patients with and without prior RG-101 dosing. *Antiviral Research*.

[118] Yamagiwa Y., Asano M., Kawasaki Y. (2016). Pretreatment serum levels of interferon-gamma-inducible protein-10 are associated with virologic response to telaprevir-based therapy. *Cytokine*.

[119] Sung P. S., Lee E. B., Park D. J. (2018). Interferon-free treatment for hepatitis C virus infection induces normalization of extrahepatic type I interferon signaling. *Clinical and Molecular Hepatology*.

[120] Gentile I., Borgia F., Buonomo A., Zappulo E., Castaldo G., Borgia G. (2014). ABT-450: a novel protease inhibitor for the treatment of hepatitis C virus infection. *Current Medicinal Chemistry*.

